# HRR gene promoter methylation-guided nomogram predicts PARP inhibitor efficacy and progression-free survival in high-grade serous ovarian cancer

**DOI:** 10.1186/s13148-026-02159-0

**Published:** 2026-05-19

**Authors:** Zhihua Zhou, Baoqin Ge, Tingting Chen, Shizhen Shen, Nalan Tu, Yuanming Shen

**Affiliations:** 1 Department of Gynecologic Oncology, Women’s Hospital, Zhejiang University School of Medicine, 1 Xueshi Road, Hangzhou, 310006 Zhejiang Province China; 2 Department of Gynecology, Hangzhou Fuyang Women and Children Hospital, 25 Hengliangting Road, Fuchun Subdistrict, Hangzhou, 311400 Zhejiang Province China

**Keywords:** High-grade serous ovarian cancer, PARP inhibitors, Homologous recombination repair, DNA methylation, Pyrosequencing, Prognostic biomarker

## Abstract

**Background:**

High-grade serous ovarian cancer (HGSOC) remains a lethal gynecologic malignancy with limited tools for accurately predicting PARP inhibitor (PARPi) efficacy. While promoter methylation of homologous recombination repair (HRR) genes is a potential biomarker, its clinical utility remains underexplored. This study aimed to systematically evaluate HRR gene promoter methylation profiles to predict PARPi sensitivity in HGSOC patients.

**Methods:**

We conducted a retrospective analysis of 96 HGSOC patients receiving PARPi maintenance therapy (olaparib or niraparib). The promoter methylation status of 12 HRR genes was quantitatively assessed via pyrosequencing. Patients were stratified into PARPi-sensitive and -insensitive groups based on OReO/ENGOT-ov38 criteria. Differential methylation sites were identified, lasso and multivariate logistic regression identified key methylation sites to construct a nomogram and a PARPi Resistance Score (PRS). PFS was analyzed by Kaplan‑Meier and Cox regression.

**Results:**

The PARPi-insensitive group exhibited significantly higher median methylation levels across HRR gene promoters compared to the sensitive group. Through rigorous screening, 12 CpG sites were consistently associated with PARPi response across subgroups. Through screening and LASSO regression, five CpG sites—ATM_Pos1, BRCA1(2S)_Pos2, BRCA2(1S)_Pos1, BRCA2(1S)_Pos7, and RAD51D_Pos6—were identified as independent predictors of PARPi sensitivity. The logistic model based on these five sites showed good discrimination, with area under the ROC curve (AUC) of 0.847 in the training set and 0.903 in the validation set; calibration and decision curve analysis supported clinical utility. Using the optimal PRS cutoff of − 1.023, High-PRS patients had significantly shorter median PFS (14 months vs. not reached; log‑rank *P* < 0.0001) and lower 12‑month PFS rate (52% vs. 97.2%). Multivariate Cox analysis confirmed PRS as the only independent prognostic factor for PFS (HR 4.85, 95% CI 2.31–10.20, *P* < 0.001).

**Conclusion:**

HRR gene promoter methylation, particularly at specific BRCA1, BRCA2 and RAD51D CpG sites, robustly predicts PARPi sensitivity and independently stratifies PFS in HGSOC patients. The nomogram and PRS provide clinically actionable tools for individualized PARPi therapy.

**Supplementary Information:**

The online version contains supplementary material available at 10.1186/s13148-026-02159-0.

## Introduction

High-grade serous ovarian cancer (HGSOC) remains the most lethal gynecological malignancy, posing a significant challenge in clinical management [1]. Despite initial responses to cytoreductive surgery and platinum-based chemotherapy, approximately 70% of patients experience relapse [2, 3]. Accumulating clinical evidence has demonstrated that maintenance therapy with poly(ADP-ribose) polymerase inhibitors (PARPis) significantly prolongs progression-free survival (PFS) in HGSOC patients with deleterious genetic variants in homologous recombination repair (HRR) genes [4, 5]. Homologous recombination deficiency (HRD) is a cellular state characterized by impaired HRR function, leading to genomic instability and increased sensitivity to PARPis [6, 7].

Current clinical HRD testing encompasses three main categories: HRR gene mutation testing, genomic scar assessment (e.g., loss of heterozygosity, telomeric allelic imbalance, large-scale state transitions), and functional assays [8–11]. Although the FDA has approved genomic scar-based kits such as Myriad myChoice® CDx and FoundationFocus™ CDx BRCA LOH for commercial use [12], their routine clinical application remains limited due to sample requirements, assay complexity, and incomplete capture of all HRD mechanisms. Moreover, some patients without detectable genomic scars still derive benefit from PARPis, particularly those with hypermethylation of the BRCA1 or RAD51C promoter. DNA methylation, especially hypermethylation of CpG islands within gene promoters, provides an important basis for early diagnosis, prognosis evaluation, treatment selection, and monitoring of tumor recurrence. Methylation testing can identify patients with epigenetic silencing of HRR genes even in the absence of BRCA mutations, potentially expanding the use of PARPis to a broader HRD population.

However, reports on the use of methylation detection to predict PARPi sensitivity in ovarian cancer remain scarce, and the clinical validity of BRCA1 or RAD51C promoter methylation for this purpose has yielded conflicting results [13–15]. These discrepancies are partly attributable to technical limitations in measuring tumor DNA methylation and differences in analytical validity. Various techniques are available for DNA methylation analysis, including methylation-specific PCR (MSP), bisulfite sequencing (BS-Seq), methylation microarrays, targeted methylation sequencing, and methylation-specific digital PCR (ddPCR). These methods, however, face limitations in quantification breadth, technical complexity, and cost efficiency. In contrast, pyrosequencing has emerged as a superior alternative for clinical applications because of its unique advantages: simultaneous quantification of adjacent CpG sites with real-time monitoring, rapid turnaround (within 15 min), significantly lower cost, and a streamlined workflow requiring minimal post-analysis steps [16]. These attributes increase its accessibility and cost-effectiveness for routine clinical use.

In this study, we aimed to systematically investigate the promoter methylation status of a comprehensive panel of HRR genes—including ATM, BRCA1, BRCA2, BRIP1, CHEK2, PALB2, RAD50, RAD51, RAD51B/C/D, and BRAD1—in HGSOC using pyrosequencing. By comparing methylation profiles between PARPi-sensitive and PARPi-insensitive patients, we sought to identify differentially methylated CpG sites associated with treatment response and to develop a predictive model for PARPi efficacy. Ultimately, we established a PARPi Resistance Score (PRS) based on key methylation sites, which may serve as a clinically useful tool to refine patient selection for PARPi maintenance therapy.

## Materials and methods

### Patients and samples

Eligible patients included those who had newly diagnosed or platinum-sensitive relapsed HGSOC patients who received olaparib (300 mg twice daily) or niraparib (200 mg once daily) tablets as first-line or platinum-sensitive relapse maintenance therapy within 8 weeks of receiving their last dose of chemotherapy at the Gynecological Oncology Center of Women’s Hospital School of Medicine Zhejiang University, China, from 2019 to 2022. All the patients received standard initial treatment (including debulking surgery combined with adjuvant platinum-based chemotherapy) and postrelapse treatment (including platinum-based chemotherapy ± cytoreductive surgery) for HGSOC and achieved a complete response or partial response. First-line maintenance therapy was limited to patients with advanced stage III-IV disease (FIGO), and recurrent maintenance therapy was not limited to patients at the initial diagnosis. There was no limit to the number of prior lines of chemotherapy patients could have received, but the most recent line of chemotherapy was not less than four cycles of platinum-based chemotherapy. Patients were required to receive any kind of PARPi maintenance therapy but only once. The follow-up was last updated in May 2024.

All samples were collected from patients who provided informed consent. In total, 96 primary tumor tissue and matched blood samples were taken before the initial treatment, 89 samples were from resected specimens of patients who underwent primary debulking surgery (PDS), and 7 samples were taken from biopsied samples of patients who received neoadjuvant chemotherapy plus interval debulking surgery (IDS).

This study followed the World Medical Association Declaration of Helsinki recommendations and was approved by the Ethics Committee of Womens Hospital School of Medicine Zhejiang University (PRO2025-219).

### Testing for BRCA genes and HRD status

BRCA1 and BRCA2 gene mutations were analyzed via next-generation sequencing (NGS). If the BRCA gene test (germline or somatic) is negative, further HRD testing will be conducted. HRD status was assessed via histological slides and evaluated via comprehensive genomic scar analysis, integrating next-generation sequencing (NGS) to assess loss of heterozygosity (LOH), telomeric allelic imbalance (TAI), and large-scale state transitions (LSTs). The threshold for the genomic instability score (GIS) was set at 42 according to the guidelines provided by the assay’s producer(Myriad myChoice® CDx).

### HRR gene promoter methylation test

Representative tumor blocks with > 70% tumor cellularity and low necrosis content were subjected to tumor DNA extraction, which two HGSOC-dedicated pathologists confirmed. Approximately 100–200 ng of genomic DNA was bisulfite-converted with the EZ DNA Methylation Kit (Qiagen Research, 51,304). Methylation treatment was performed via the Qiagen EpiTect Bisulfite Kit (Qiagen 59,104). The primer design software used was PyroMark Assay Design 2.0, and the primer sequence information can be found in supplementary S1. HRR gene promoter methylation was determined by pyrosequencing (QIAGEN, Q48).

### PARPi sensitivity definition

To establish a clinically meaningful and reproducible definition of PARPi response, we adopted the efficacy thresholds used in the phase IIIb OReO/ENGOT-ov38 trial, a study specifically designed to evaluate PARPi rechallenge [17]. This definition operationalizes “sensitivity” as deriving a substantial and durable clinical benefit from maintenance therapy, which is pragmatically reflected by the duration of treatment before discontinuation due to progression or toxicity.

Specifically, patients were classified as PARPi-sensitive if they maintained treatment without disease progression for:⑴ ≥ 18 months following first-line chemotherapy, or ≥ 12 months following second-line or later chemotherapy, if they harbored a BRCA mutation; ⑵ ≥ 12 months following first-line chemotherapy, or ≥ 6 months following second-line or later chemotherapy, if they were BRCA wild-type.

Patients who discontinued PARPi therapy before meeting these time thresholds were classified as PARPi-insensitive. This dichotomous endpoint is a recognized and clinically relevant surrogate for prolonged progression-free survival in retrospective analyses of PARPi maintenance therapy, as it identifies the cohort with the most sustained disease control.

### Follow-up and endpoints

Patients were followed every 3 months for the first 2 years, every 6 months for years 3–5, and annually thereafter. Evaluations included medical history, physical examination, serum CA125, and radiological imaging. Disease progression was assessed using RECIST 1.1 criteria [18]. Progression-free survival (PFS) was calculated from the initiation of PARPi maintenance therapy to the date of disease progression, recurrence, or death.

### Statistical analysis

Statistical analysis was performed with SPSS for Windows (version 26.0, SCR_002865) and R software (R Foundation for Statistical Computing). Differences between two independent groups were tested with the Mann‒Whitney U test (continuous variables and nonparametric analyses). For categorical variables, the Chi-square test or Fisher's exact test was used as appropriate. Receiver operating characteristic (ROC) curves were constructed to assess the sensitivity, specificity, and respective areas under the curves (AUCs) with 95% CIs. We investigated the optimum cutoff value for diagnosis by the Youden index (sensitivity + specificity-1) and by minimizing the cutoff value's distance to the ROC curve's top-left corner. LASSO regression with tenfold cross-validation followed by multivariable logistic regression identified independent methylation predictors of PARPi sensitivity; a nomogram was constructed accordingly. Model performance was evaluated by AUC, calibration curves (Brier score, Hosmer–Lemeshow test), and decision curve analysis. The PARPi Resistance Score (PRS) was derived from the linear predictor of the model. Survival curves were estimated using the Kaplan–Meier method and compared by the log-rank test. Multivariate Cox proportional hazards regression was used to evaluate independent prognostic factors for PFS. A two-sided *P* < 0.05 was considered statistically significant.

## Results

### Study population

The baseline clinicopathological and genetic characteristics of the 96 patients are summarized in Table 1. According to the OReO/ENGOT-ov38 study criteria, 78 patients were classified as PARPi-sensitive, and 18 patients were classified as PARPi-insensitive. The median ages of the patients in the PARPi-sensitive and insensitive groups were 55 and 54.5 years, respectively (Mann–Whitney U test, *p* = 0.59). Most patients received PARPi first-line maintenance therapy (76/96, 79.17%), and 20.83% (20/96) received PARPi platinum-sensitive relapse maintenance therapy. A total of 7 patients (7/96, 7.29%) received neoadjuvant chemotherapy followed by IDS, but the majority received PDS and adjuvant chemotherapy. The primary complete cytoreductive surgery (R0) rate was 79.17% (76/96). A total of 80 patients were subjected to genetic testing, 74 of whom were HRD positive, 56 of whom had germline or somatic BRCA mutations, and 6 of whom were HRD negative. Among the 16 patients who did not undergo HRD testing, 12 voluntarily opted out as they met the clinical criteria for PARPi maintenance therapy due to platinum-sensitive recurrence; the remaining 4 declined HRD testing due to personal preference and only consented to BRCA testing. The comparison between the two groups revealed no significant differences in age at onset, tumor stage, presence of ascites, ideal cytoreduction, timing of PARPi administration, type of PARPi used, or genetic status (*P* > 0.05). However, there was a specific difference between PDS and IDS (*P* = 0.028 < 0.05).

**Table 1:** Patient characteristics (clinical, pathological, and molecular)

	PARPi sensitive (N = 78)	PARPi insensitive (N = 18)	*P*
Age			
Median (range)	55 (48–62)	54 (46–65)	0.59
FIGO stage			
Ⅰ/Ⅱ	8	1	0.69
Ⅲ	58	15	
Ⅳ	12	2	
Ascites			
Yes	57	15	1.00
No	11	3	
Surgical options			
PDS	75	14	0.03
IDS	3	4	
Cytoreductive surgery			
R0	63	13	0.63
No R0	15	5	
PARPi lines			
Fist-line	63	13	0.63
Recurrent	15	5	
PARPi type			
Olaparib	35	7	0.65
Niraparib	43	11	
BRCA mutation			
Wild type	26	8	0.68
BRCA +	47	9	
Undetermined	5	1	
HRD status			
HRD-	6	0	0.14
HRD +	61	13	
Undetermined	11	5	

### Target methylation site screening

Twelve HRR-related genes (ATM, BRAD1, BRCA1/2, BRIP1, CHEK2, PALB2, RAD50, RAD51, RAD51B/C/D) were included in the preliminary screening, with 1–3 primers designed for each gene, resulting in 214 methylation sites being used for the preliminary screening (Table 2, Supplementary Table S1). We randomly selected 28 patients (14 PARPi-sensitive patients and 14 PARPi-insensitive patients) for pyrosequencing and compared the methylation rates between the two groups. The baseline characteristics of these 28 patients are presented in Supplementary Table S2 and were balanced between groups (all *P* > 0.05). The mean differences at each CpG site between the two groups revealed 42 CpG sites with differential methylation rates exceeding 15%, which were associated with 10 HRR genes and 17 primers (Supplementary Table S3). Ultimately, primers harboring ≥ 2 differentially methylated CpG sites, including ATM, BRAD1, BRCA1, BRCA2, CHEK2, PALB2, RAD51, and RAD51D (Table 3), were selected as candidates for further analysis. Finally, 10 primers and 96 candidate methylation sites were used for subsequent analysis (Table 3, Supplementary Table S4).

**Table 2:** Preliminary screening of HRR promoter methylation sites

Gene	Number of primers	Number of methylation sites
ATM	2	15
BRAD1	3	24
BRCA1	3	23
BRCA2	3	31
BRIP1	1	5
CHEK2	2	16
PALB2	3	25
RAD50	2	14
RAD51	2	14
RAD51B	1	5
RAD51C	3	27
RAD51D	2	15
Total	27	214

**Table 3:** Candidate HRR promoter methylation sites

Gene	Number of primers	Number of methylation sites
ATM	1	8
BRAD1	1	13
BRCA1	2	15
BRCA2	2	24
CHEK2	1	11
PALB2	1	9
RAD51	1	9
RAD51D	1	7
Total	10	96

### HRR gene methylation in PARPi-sensitive and PARPi-insensitive EOC

**Fig. 1:**
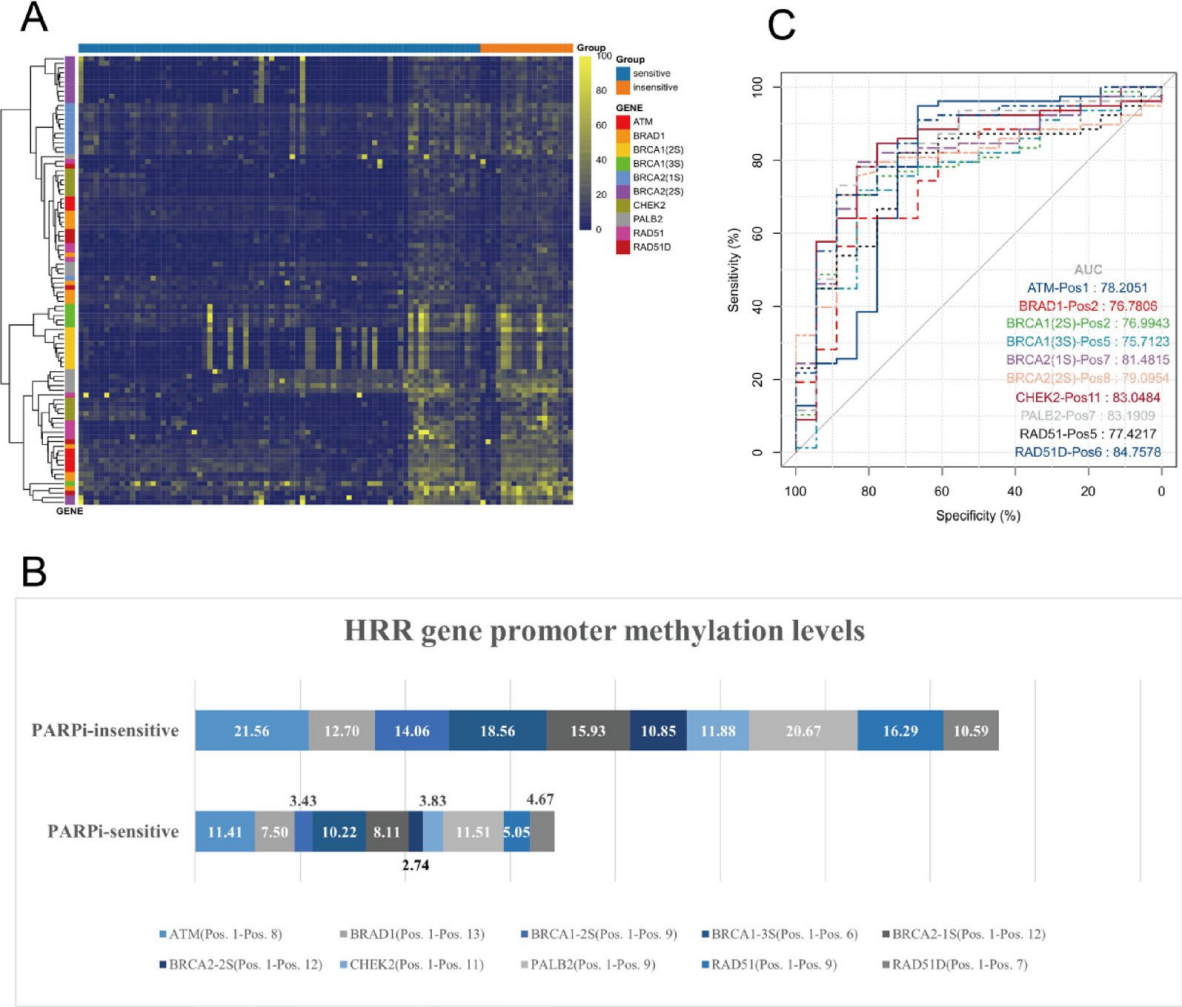
Comparison of HRR gene promoter methylation levels between the PARPi-sensitive and PARPi-insensitive groups. **A** Heatmap features of the two clusters based on the methylation patterns of the HRR genes. **B** Median cumulative methylation level of the HRR gene promoter region. **C** ROC curves for the maximum AUC of each HRR gene locus

Then, 10 primers for 8 HRR genes, ATM, BRAD1, BRCA1, BRCA2, CHEK2, PALB2, RAD51, and RAD51D, were used for pyrosequencing detection in paraffin-embedded tumor tissue from those 96 patients (Fig. 1A). Methylation detection revealed that the median methylation rate of the HRR gene promoters in the PARPi-insensitive group was significantly greater than that in the sensitive group (Fig. 1B). The methylation levels at multiple sites within the corresponding promoter regions of each HRR gene were significantly different between the PARPi-sensitive and PARPi-insensitive groups. Compared with the insensitive group, there were no significant differences in methylation status at the following sites: BRAD1 Pos7 and Pos10 or BRCA1 (primer 3S) Pos2. Overall, the methylation levels at the remaining 93 sites were generally significantly lower in the PARPi-sensitive group than in the insensitive group, with significant differences between the two groups (*P* < 0.05, Supplementary Figure S1/Table S5).

**Fig. 2:**
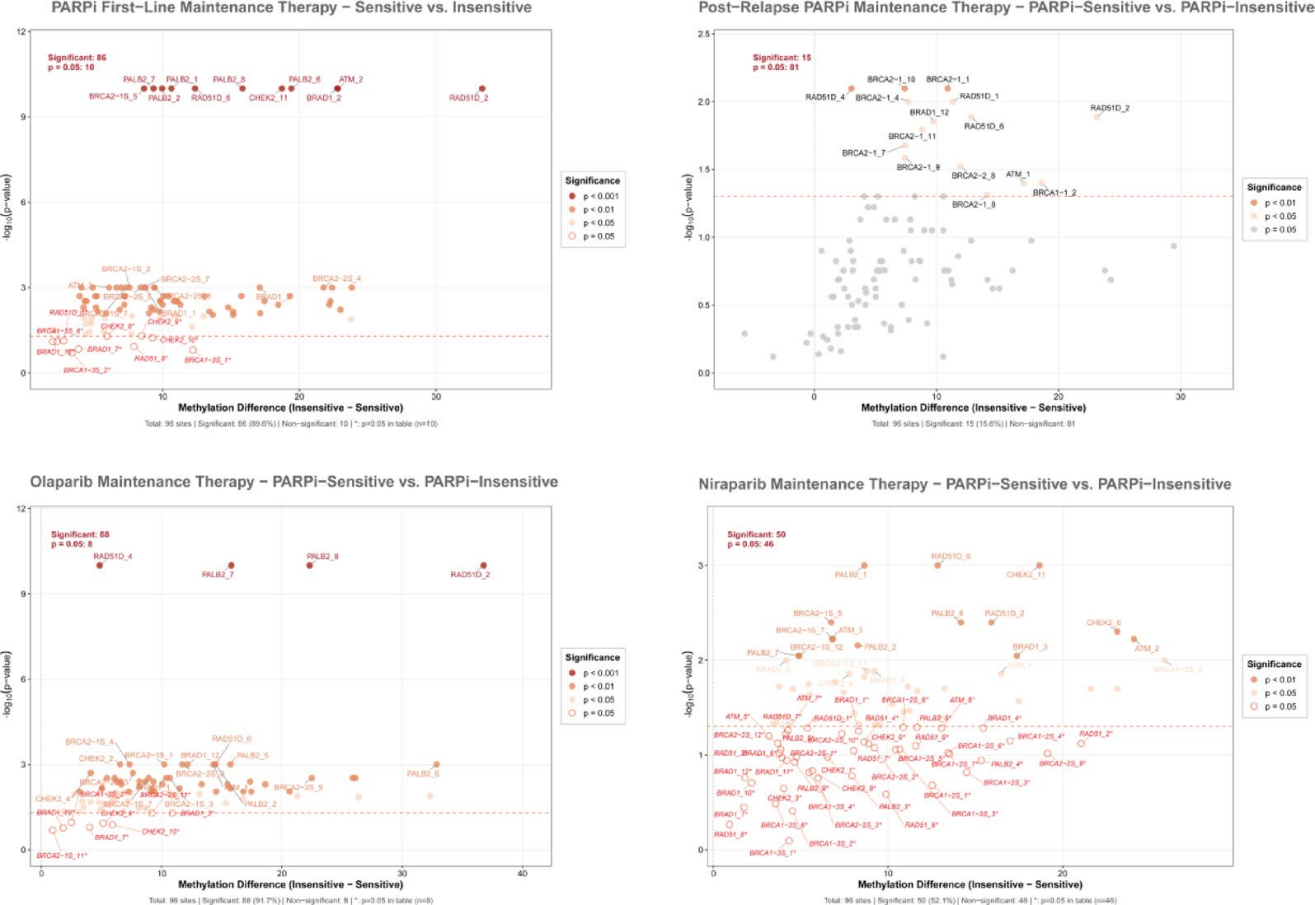
Volcano plot of HRR gene promoter methylation differences between PARPi-sensitive and -insensitive groups. **A** First-line PARPi maintenance subgroup. **B** Relapsed PARPi maintenance subgroup. **C** Olaparib maintenance subgroup. **D** Niraparib maintenance subgroup.The x-axis shows the methylation difference (Insensitive – Sensitive, %). The y-axis represents statistical significance (-log₁₀ *p*-value). The dashed line indicates *P* = 0.05

Subgroup analysis of the differences in the HRR methylation levels between the PARPi-sensitive patients and insensitive patients receiving first-line maintenance therapy revealed that out of the 76 patients receiving PARPis as first-line maintenance therapy, 63 were PARPi-sensitive, and 13 were insensitive. Among the 96 HRR promoter methylation sites, 86 were significantly different. The methylation levels were significantly lower in the PARPi-sensitive group than in the insensitive group (adjusted *P* < 0.05, Fig. 2A). Further analysis of patients who received PARPis after recurrence revealed that 15 patients were sensitive to PARPis and that 5 patients were insensitive. Among the 96 HRR promoter methylation sites, only 15 sites were significantly different, with lower methylation levels in the recurrent maintenance-sensitive group than in the insensitive group (adjusted *P* < 0.05, Fig. 2B). Additional subgroup analyses were performed. In patients receiving olaparib (n = 42), 88 methylation sites were significantly different between sensitive and insensitive groups, while in the niraparib group (n = 54), 50 sites showed significant differences (Fig. 2C, D).

In our research, we sought to comprehensively evaluate the utility of methylation levels in the promoter regions of homologous recombination repair (HRR) genes as biomarkers for assessing the therapeutic efficacy of PARPis in EOC patients. On the basis of the final sensitivity outcomes of PARPi treatment, we computed the area under the curve (AUC) for each methylation site, and the optimal cutoff values for each methylation site in predicting PARPi sensitivity were determined via Youden’s index (Supplementary Table S6). The ROC curves for the maximum AUC of each HRR gene locus are shown in Fig. 1C. The higher AUC suggests that the HRR gene promoter methylation level could serve as an effective diagnostic tool for predicting sensitivity to PARPi therapy in EOC patients.

### Establishment and evaluation of a prediction model for PARPi sensitivity detection via HRR promoter methylation

**Fig. 3:**
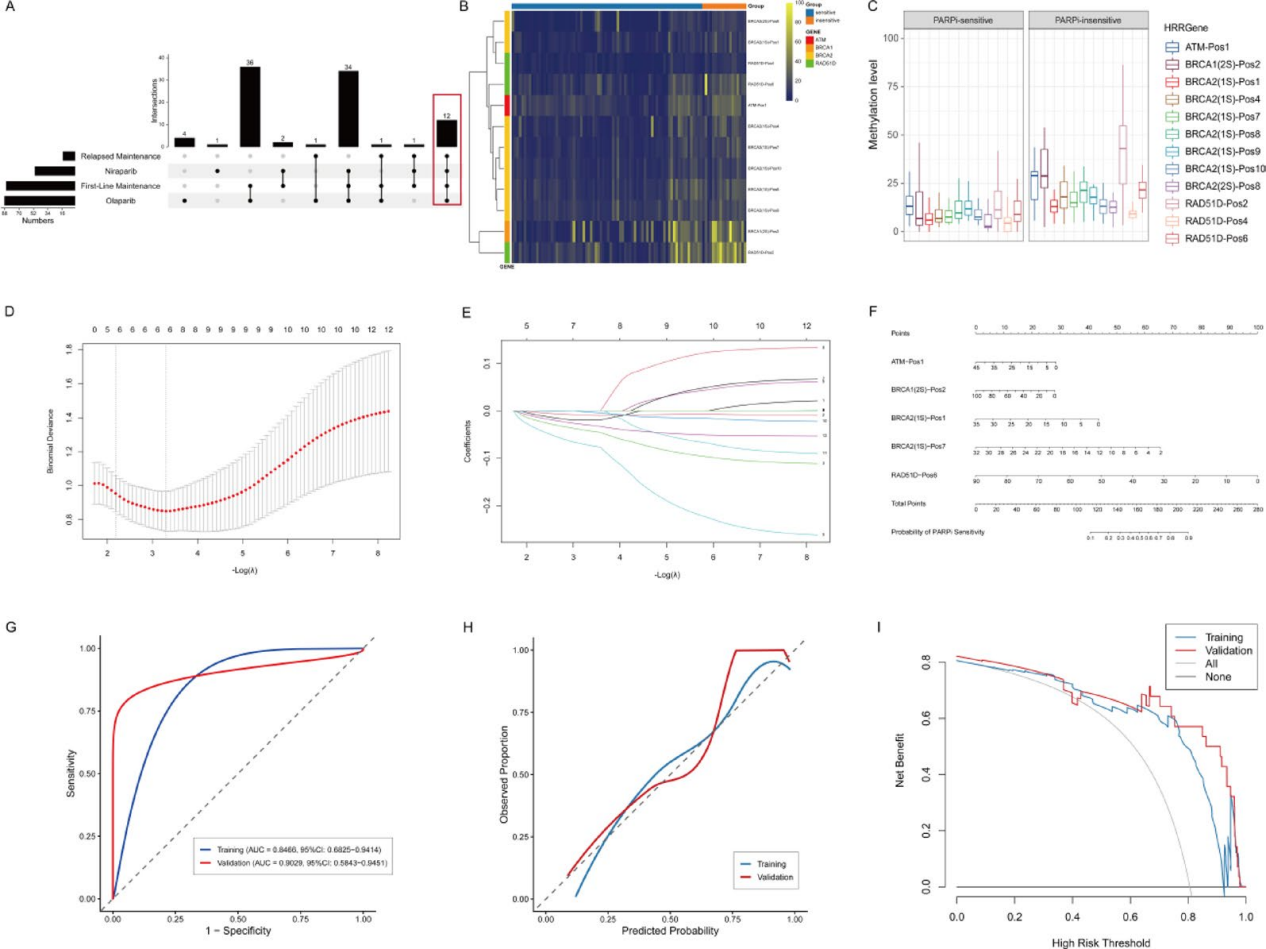
Establishment and evaluation of a prediction model for PARPi sensitivity detection via HRR promoter methylation. **A** UPSET plot showing overlap of differential methylated CpG sites across four subgroups. Twelve sites were significantly associated with PARPi sensitivity in all subgroups. **B** Heatmap showing the methylation levels of 12 CpG sites significantly associated with PARPi sensitivity. **C** Boxplots comparing methylation levels of the 12 identified CpG sites between PARPi-sensitive and -insensitive groups. Each site shows the distribution (median, quartiles, and outliers) for both groups. **D**, **E** Parameter plots for LASSO regression, identifying 6 HRR methylation sites on the basis of lambda. **F** Nomogram predicting the probability of PARPi sensitivity based on five CpG sites identified by logistic regression. Points are assigned to each site according to methylation level; the total points correspond to the predicted probability of sensitivity. **G** Constructing receiver operating characteristic (ROC) curves individually for the training and validation sets to assess the accuracy of the model. **H** Calibration curves of the nomogram for predicting PARPi sensitivity in the training and validation sets **I** A clinical decision curve was constructed to evaluate the clinical practicality of the prediction model

Based on the aforementioned subgroup analysis, we identified 12 promoter methylation sites across four HRR genes that exhibited statistically significant differences (*P* < 0.05) in all four subgroups (Fig. 3A–C). These 12 sites exhibited strong predictive performance (Supplementary Table S7). To further develop a predictive model linking HRR gene promoter methylation levels with the efficacy of PARP inhibitor therapy, 96 patients were divided into training and validation groups at an approximately 3:1 ratio, with 68 in the training group and 28 in the validation group. LASSO logistic regression with tenfold cross‑validation on the training set initially selected six predictors: ATM‑Pos1, BRCA1(2S)‑Pos2, BRCA2(1S)‑Pos1, BRCA2(1S)‑Pos7, RAD51D‑Pos4, and RAD51D‑Pos6. RAD51D‑Pos4 was excluded from the final model because its coefficient was negligible and its removal did not affect model performance (AIC: 54.89 vs. 56.89; C‑index: 0.8647 vs. 0.8661). The final multivariable logistic regression model based on the remaining five variables yielded the prediction formula: logit(P) = 4.8304 − 0.0284 * ATM.Pos1 − 0.0126 * BRCA1.2S.Pos2 − 0.0560 * BRCA2.1S.Pos1 − 0.0985 * BRCA2.1S.Pos7 − 0.0501 * RAD51D.Pos6, where P is the probability of PARPi sensitivity. A nomogram was constructed accordingly (Fig. 3D–F). The final model showed good discrimination with an AUC of 0.847 (95% CI 0.683–0.941) in the training set and 0.903 (95% CI 0.584–0.945) in the validation set (Fig. 3G). Calibration was also satisfactory, with Brier scores of 0.0953 and 0.0893, and Hosmer Lemeshow p values of 0.7856 and 0.5149 for the training and validation cohorts, respectively (Fig. 3H). Decision curve analysis showed that the model’s net benefit exceeded that of the ‘treat all’ and ‘treat none’ strategies across a wide range of clinically relevant threshold probabilities in both the training and validation cohorts, indicating its potential clinical utility for guiding PARPi therapy(Fig. 3I). The nomogram derived from this model serves as a practical tool for individualized prediction of the likelihood of benefit from PARP inhibitor therapy.

### Association of the PRS with progression-free survival and prognostic stratification

To translate the predictive nomogram into a clinically actionable tool, we established a continuous metric termed the PARPi Resistance Score (PRS). This score was calculated using the linear predictor from the logistic regression model: PRS = − 4.8304 + 0.0284 * ATM.Pos1 + 0.0126 * BRCA1.2S.Pos2 + 0.0560 * BRCA2.1S.Pos1 + 0.0985 * BRCA2.1S.Pos7 + 0.0501 * RAD51D.Pos6. Conceptually, higher PRS values correspond to a greater likelihood of PARPi resistance. Using the optimal cutoff of − 1.023 determined by the Youden index, the cohort was stratified into a High-PRS group (PRS > − 1.023, indicating high risk of resistance) and a Low-PRS group (PRS < − 1.023, indicating sensitivity).

**Fig. 4:**
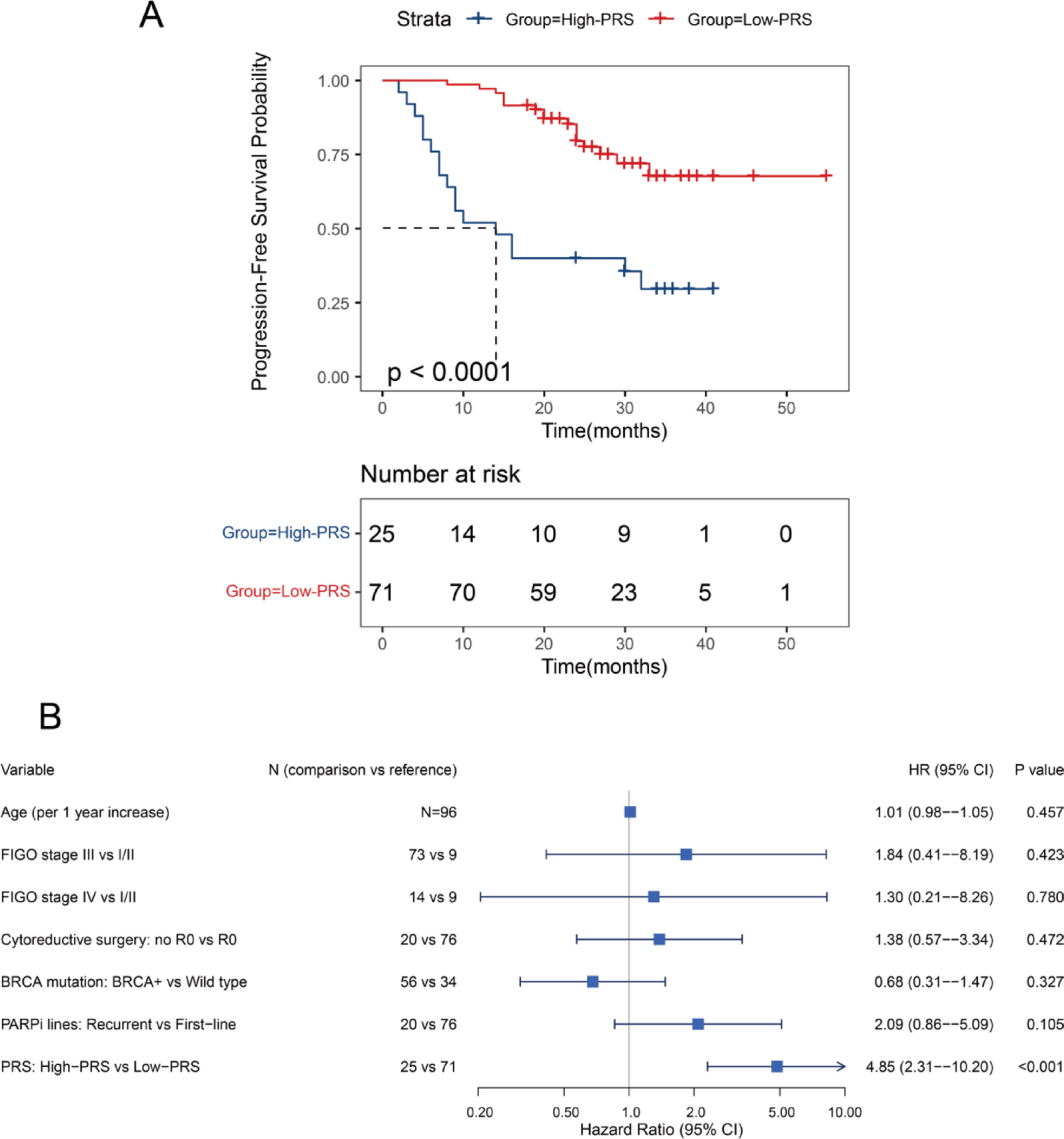
Prognostic stratification of the PRS and independent survival analysis. **A** Kaplan–Meier curves for progression-free survival (PFS) in the overall cohort stratified by the PRS. **B** Forest plot of the multivariate Cox proportional hazards regression analysis for PFS

Kaplan–Meier survival analysis revealed a compelling separation of PFS curves in the overall cohort (Fig. 4A). Patients in the High-PRS group experienced a drastically shorter median PFS compared to those in the Low-PRS group (14 months vs. not reached; log-rank P < 0.0001). The 12-month progression-free survival rate was markedly lower in the High-PRS group (52% vs. 97.2%), underscoring the clinical relevance of this stratification.

Furthermore, multivariate Cox proportional hazards regression analysis was performed to validate the independent prognostic value of the PRS. After adjusting for a comprehensive panel of clinical variables—including age, FIGO stage, surgical approach (PDS vs. IDS), residual disease status, BRCA mutation status, and therapeutic setting—the PRS remained the only independent prognostic factor for PFS. Patients in the High-PRS group exhibited a significantly increased risk of disease progression or recurrence compared to the Low-PRS group (hazard ratio [HR] 4.85; 95% confidence interval [CI] 2.31–10.20; *P* < 0.001). Notably, none of the conventional clinical parameters, including FIGO stage and BRCA mutation status, reached statistical significance in the multivariate model (all *P* > 0.05) (Fig. 4B). These findings strongly suggest that the PRS serves as a robust, independent biomarker for long-term survival in patients treated with PARPi, offering superior prognostic information compared to traditional clinicopathological factors.

## Discussion

This study provides comprehensive evidence that promoter methylation levels of homologous recombination repair (HRR) genes are significantly associated with PARP inhibitor (PARPi) sensitivity in high-grade serous ovarian cancer (HGSOC) patients. Through systematic screening of 214 CpG sites across 12 HRR genes and subsequent validation in 96 patients, we identified that patients in the PARPi-insensitive group exhibited significantly higher methylation levels across multiple HRR gene promoters compared to those in the sensitive group. Notably, this differential methylation pattern was particularly pronounced in patients receiving first-line PARPi maintenance therapy and in those treated with olaparib. Through rigorous subgroup analysis and lasso regression modeling, we identified five key hypermethylation sites—ATM_Pos1, BRCA1(2S)_Pos2, BRCA2(1S)_Pos1, BRCA2(1S)_Pos7, RAD51D_Pos6—as independent predictors of PARPi resistance. A nomogram incorporating these five sites demonstrated excellent predictive accuracy in training and validation sets. Furthermore, we established a PARPi Resistance Score (PRS) based on these sites, which served as the only independent prognostic factor for progression-free survival (PFS) in multivariate Cox regression analysis (HR 4.85, *P* < 0.001), outperforming traditional clinical parameters including FIGO stage, surgical outcome, and BRCA mutation status.

Our finding that higher methylation levels are associated with PARPi resistance appears counterintuitive to the classical model, wherein promoter hypermethylation leads to gene silencing and homologous recombination deficiency (HRD), thereby theoretically conferring PARPi sensitivity through synthetic lethality. However, several lines of evidence support a more complex relationship between methylation status and therapeutic response. First, the observed hypomethylation in the sensitive group may reflect a functionally intact HRR pathway with transcriptionally active genes maintaining baseline DNA repair capacity. In this context, PARPi efficacy may not operate primarily through canonical synthetic lethality but rather via HRD-independent cytotoxic mechanisms, particularly the trapping of PARP-DNA complexes [19–21]. Second, hypermethylation in the insensitive group may not be solely linked to HRD but could instead represent a broader epigenetic dysregulation associated with aggressive tumor biology and multifaceted resistance mechanisms. Emerging evidence suggests that PARPi resistance evolves through coordinated epigenetic remodeling: silencing of HRR genes may reshape the DNA damage response by activating error-prone repair pathways (e.g., NHEJ/MMEJ), while methylation-driven alterations in drug disposition genes can establish pharmacokinetic barriers [22–24]. These processes further interface with pro-survival signaling networks, particularly hyperactivated PI3K/AKT/mTOR pathways, to collectively establish an epigenetically primed survival state [25–28]. Notably, our findings align with sporadic reports, including a study by Félix Blanc-Durand et al., which identified patients with hypomethylated tumors who demonstrated high genomic instability scores (GIS) and, despite limited follow-up, showed no recurrence, suggesting alternative mechanisms underlying PARPi sensitivity beyond conventional methylation-driven HRD pathways [29].

The discrepancies between our findings and studies reporting hypermethylation-induced PARPi sensitivity may also stem from methodological and systemic differences. Critical variables include the specificity of detection techniques, the selection of promoter regions analyzed, the panel of HRR genes examined, and the definition of PARPi response [30]. To address the inherent heterogeneity in methylation detection methodologies, we selected pyrosequencing as our analytical platform. This approach offers distinct advantages: compatibility with FFPE tissues through small amplicon analysis that circumvents DNA degradation challenges, and quantitative assessment of individual CpG sites within specific regions, thereby improving resolution and minimizing interpretation bias. Furthermore, our study directly evaluated the relationship between HRR gene methylation status and clinical outcomes rather than relying on indirect prognostic inference from surrogate biomarkers such as GIS [31, 32]. This approach minimizes confounding biases inherent in predictive modeling and establishes a more direct causal linkage between methylation patterns and observed treatment responses.

The clinical utility of our predictive model is substantial. To our knowledge, this is the first predictive nomogram for PARPi sensitivity that incorporates genome-wide HRR gene methylation profiles developed from a real-world clinical cohort. The nomogram, based on five easily measurable CpG sites, provides an intuitive visual tool for clinicians to estimate individual patient probability of deriving benefit from PARPi therapy. The model demonstrated excellent calibration and positive decision curve analysis, indicating reliable clinical utility across a range of threshold probabilities. Furthermore, the PRS derived from this model successfully stratified patients into distinct prognostic groups: the high-PRS group experienced significantly shorter median PFS, highlighting its potential as a complementary biomarker to existing testing strategies. This suggests that the methylation status of HRR genes may capture a subset of patients with occult resistance mechanisms who might otherwise be missed by standard genomic testing.

A critical distinction of our study is its comprehensive evaluation of multiple HRR genes beyond BRCA1/2. While most previous investigations have focused solely on BRCA gene methylation [29, 31, 33], our analysis encompassed 12 HRR genes and ultimately identified key predictive sites in BRCA2 and RAD51D. This multi-locus strategy mitigates the risk of false negatives inherent to single-gene approaches and captures a broader spectrum of epigenetic dysregulation relevant to PARPi response. The inclusion of RAD51D, a key component of the homologous recombination machinery, is particularly noteworthy given its established role in DNA repair and emerging evidence linking RAD51 family member methylation to therapeutic outcomes [34, 35].

Several limitations of this study should be acknowledged. First, the relatively small sample size, particularly in the PARPi-insensitive group (n = 18), may limit the generalizability of our findings and precluded external validation of the predictive model. Second, incomplete HRD testing data for some patients (16/96) prevented a comprehensive head-to-head comparison between methylation-based and HRD-based predictive approaches. Third, the retrospective design introduces potential selection bias, and the binary definition of PARPi sensitivity based on treatment duration thresholds, while clinically relevant and derived from established trial criteria (OReO/ENGOT-ov38), may not fully capture the continuum of therapeutic benefit. Fourth, significant differences in baseline characteristics between PDS and IDS cohorts (*p* = 0.03) were observed, which may reflect underlying biological differences in treatment-naïve versus chemotherapy-exposed tumors. This finding aligns with biomarker analyses from the PAOLA-1 trial, which demonstrated superior olaparib benefit in patients receiving ≤ 1 cycle of neoadjuvant chemotherapy (HR 0.48) versus those receiving > 1 cycle (HR 0.76), suggesting that treatment-naïve tumors may better retain therapeutic vulnerabilities [36].

Future prospective studies with larger, multi-institutional cohorts, complete HRD and BRCA testing data, and standardized PFS/OS endpoints are warranted to validate our findings and refine the predictive model. Additionally, functional studies exploring the mechanistic links between site-specific methylation patterns and resistance mechanisms would provide biological validation of our clinical observations. Integration of our methylation-based PRS with other emerging biomarkers—such as genomic scar scores, mutational signatures, and immune microenvironment profiles—may ultimately enable a composite biomarker strategy for optimal patient selection for PARPi therapy.

## Conclusions

In conclusion, this study demonstrates that HRR gene promoter methylation patterns, particularly at specific BRCA1、BRCA2 and RAD51D CpG sites, are significantly associated with PARPi sensitivity and serve as independent prognostic factors for PFS in HGSOC patients. The developed nomogram and PRS provide clinically actionable tools for individualizing treatment decisions and identifying patients most likely to derive durable benefit from PARPi maintenance therapy.

## Supplementary Information

Below is the link to the electronic supplementary material.

Supplementary Material 1.

## Data Availability

All data generated or analyzed during this study are included in this article and supplementary materials. Raw data from this study are available from the corresponding author upon reasonable request.

## Abbreviations

AUC: Area Under the Curve
BRCA: Breast Cancer gene
CI: Confidence Interval
CpG: Cytosine-phosphate-Guanine
DCA: Decision Curve Analysis
ddPCR: Droplet Digital Polymerase Chain Reaction
EOC: Epithelial Ovarian Cancer
FFPE: Formalin-Fixed Paraffin-Embedded
FIGO: International Federation of Gynecology and Obstetrics
GIS: Genomic Instability Score
HGSOC: High-Grade Serous Ovarian Cancer
HR: Hazard Ratio
HRD: Homologous Recombination Deficiency
HRR: Homologous Recombination Repair
IDS: Interval Debulking Surgery
LOH: Loss of Heterozygosity
LST: Large-Scale State Transition
MSP: Methylation-Specific PCR
NGS: Next-Generation Sequencing
NHEJ: Non-Homologous End Joining
OR: Odds Ratio
PARP: Poly(ADP-ribose) Polymerase
PARPi: PARP Inhibitor
PDS: Primary Debulking Surgery
PFS: Progression-Free Survival
PRS: PARPi Resistance Score
RAD: Radiation sensitive
ROC: Receiver Operating Characteristic
TAI: Telomeric Allelic Imbalance
